# The *KCNH2* Genetic Polymorphism (1956, C>T) Is a Novel Biomarker That Is Associated with CCB and α,β-ADR Blocker Response in EH Patients in China

**DOI:** 10.1371/journal.pone.0061317

**Published:** 2013-04-22

**Authors:** Fazhong He, Jianquan Luo, Zhiying Luo, Lan Fan, Yijing He, Dingliang Zhu, Jinping Gao, Sheng Deng, Yan Wang, Yuesheng Qian, Honghao Zhou, Xiaoping Chen, Wei Zhang

**Affiliations:** 1 Pharmacogenetics Research Institute, Institute of Clinical Pharmacology, Hunan Key laboratory of Pharmacogenetics, Central South University, Changsha, Hunan, P. R. C.; 2 Shanghai Institute of Hypertension, Ruijin Hospital, Shanghai Jiaotong University School of Medicine, Shanghai, P. R. C.; 3 Department of Pharmacy, Xiangya Hospital, Central South University, Changsha, Hunan, P. R. C.; Children's National Medical Center, Washington, United States of America

## Abstract

**Background:**

*KCNH2* (hERG) potassium channels have an integral role in regulating the excitability of smooth muscle cells. Some pathways driven by angiotensin II, nitric oxide and adrenergic receptors blocker are involved in modulating the properties of KCNH2 potassium channels. And these pathways are closely related to blood pressure regulation. Therefore, we hypothesized that *KCNH2* genetic polymorphisms may affect blood pressure response to the antihypertensive drug therapies.

**Materials and Methods:**

To evaluate the interactions between *KCNH2* genetic polymorphisms and individual blood pressure response to antihypertensive drugs, 370 subjects with essential hypertension (EH) were studied. In evaluating the interactions between *KCNH2* genetic polymorphisms and drug response to blood pressure, multivariable ANOVA analysis followed by Bonferroni correction were carried out.

**Results:**

There were statistically significant interactions between *KCNH2* (1956, C>T) polymorphism and DBP change (P = 0.010), MAP change (P = 0.014) on azelnidipine or nitrendipine therapy patients at the end of 6 weeks. We found that the *KCNH2* (1956,C>T) polymorphism was associated with the hypotensive effects of α,β-ADR blockers of DBP change at the end of 4 and 6 weeks' treatment in an age- and gender-dependent manner (P = 0.007 and 0.019, respectively). Similar results were also observed for changes in MAP at the end of 4 and 6 weeks (P-values were 0.035 and 0.078, respectively). While patients who received imidapril, candesartan and irbesartan therapy, no significant difference in drug response among *KCNH2*(1956,C>T) genotype was observed.

**Conclusion:**

We have reported for the first time that *KCNH2* (1956, C>T) polymorphism is associated with efficacy of antihypertensive drugs CCBs and ADR blockers, and may serve as a novel biomarker for individualized therapy for certain antihypertensive drugs.

## Introduction

Essential hypertension is a heterogeneous disorder with differing causal factors in various patients. Essential hypertension comprises 95% of all causes of hypertension. The seventh Report of the Joint National Committee defined and classified hypertension in adults [1]. The diagnosis of hypertension is made when the average of 2 or more diastolic blood pressure (DBP) measurements on at least 2 subsequent visits is ≥90 mm Hg and (or) systolic blood pressure (SBP) ≥140 mm Hg. Isolated systolic hypertension is defined as SBP ≥140 mm Hg and DBP<90 mm Hg. Hypertension remains a major modifiable risk factor for cardiovascular disease despite important advances in our understanding of its pathophysiology and the availability of effective treatment strategies.

Studies have widely reported that genetic factors are important determinants of hypertension susceptibility and the drug response. Human ether-a-go-go-related gene (h*ERG* or *KCNH2*) codes for the α subunit of the delayed-rectifier potassium channel (*I_kr_)*, and was first discovered as a cDNA homologous to *Drosophila Eag* gene screened from the human hippocampus cDNA library by Warmke and Ganetzky in 1994. *KCNH2* potassium channels are widely expressed in human cardiac and smooth muscle cells. *KCNH2* expression can also be detected in liver, pancreas, nervous and tumor tissues. The past researches have shown that functional abnormalities of *KCNH2* potassium channels are related to increased risk for QT syndromes and tumors. While some studies have mentioned that the *KCNH2* potassium channels also play a fundamental role in modulating the resting membrane potential in smooth muscles and neurons [2], [3], suggesting that *KCNH2* potassium channels may play a pivotal role in regulating the excitability of excitable cells.

Currently, many studies indicated that the nitric oxide (NO), ANG II, adrenergic receptor (ADR) blockers, L-type calcium channel blockers mediated pathways and some regulatory proteins are involved in modulating the properties of *KCNH2* channels. Taglialatela et al. [4] have reported that the reactive oxygen species can evoke down-regulation of *KCNH2* protein and decrease *I_kr_* but do not affect other potassium channels such as Beag, rDRK1, and mIRK1. In addition, NO release directly activates *I_kr_* in opossum esophagus circular muscle depending on Ca^2+^ release from the sarcoplasmic reticulum stores [5]. G protein-coupled receptors such as α-ADR (via PLC, PKA, PKC pathway [6]) and β1-ADR (via activation of Gs-adenylate cyclase-cAMP-PKA-14-3-3 pathway [7]) can regulate the expression of *KCNH2* potassium and (or) set its gating characteristics. Furthermore, cAMP/PKA/PKC also can directly or indirectly interact with *KCNH2* potassium channels [8]. Studies have observed that Ang II could increase *I_kr_* by about 30% with a time constant of approximately 30 s via AT1R in an ATP-dependent and PKC-mediated pathway; and the muscarinic agonist can occlude the effect of Ang II on *I_kr_* by enhancing *I_kr_*, suggesting that PLC-PIP2 pathway may be involved in regulating *KCNH2* potassium channels [9], [10], [11]. It is worth noting that the interaction between L-type calcium and *KCNH2* potassium channels is regulated by endothelin-1(ET-1) induced ANP secretion rather than PKC-mediated pathway [12],while small GTPase Rab11b disorders lead to an increase in L-type Ca^2+^ current but a decrease in *I_kr_* [13]. Moreover, the *KCNH2* potassium channels may directly regulate the characteristics of sodium and calcium channels [14]. Finally, some regulatory proteins such as Caveolin-1, dynamin-2 and monoubiquitination also play an important role in modulating the properties or endocytic degradation of *KCNH2* potassium channels [15], [16], [17].

Interestingly, all the pathways outlined above are closely related to the pathological process of hypertension. Antihypertensive drugs like α-, or β-ADR blocker,ACE-inhibitor, and calcium channel blockers (CCB) are also able to regulate blood pressure via these pathways. The aromatic rings of *KCNH2* Y652 and F656 located in S6 domain are the key determinants of a variety of xenobiotics (eg. doxazosin) or endogenous substances (eg. hormones) binding site [18], and the mutations Y652A (1956,C>T) and F656A (1966–1967insT) can attenuate the sensitivity of the targeting substances [19], [20]. Guo et al. [21] found that the effect of drugs on *KCNH2* channels is not necessarily to block KCNH2 channels, but through targeting the *KCNH2*-interacting proteins such as Caveolin-1, and the mutations of *KCNHE2* can influence such mutual effect [17]. *KCNH2* (2690, A>C) mutation can create a phosphorylation site, and can also result in the increase in aldosterone synthesis, indicating that this mutation may change the channels activities [22].[23]. Hence, we hypothesize that the mutations of *KCNH2* (1956, C>T, 1966–1967insT and 2690, A>C) may influence the hypotensive effects of antihypertensive drugs. In this study, a total of 370 eligible patients was studied after a run-in period of 2 weeks and assigned to receive the antihypertensive drugs for 4 weeks or more.

## Results

### 1. Baseline characteristics of patients

Genotyping by sanger-sequencing failed to find the *KCNH2* (1966–1967insT) polymorphism in 85 randomly selected patients. The *KCNH2* (2690, A>C) mutation (C allele frequency was 2.9%) was not associated with the hypotensive effects of the anti-hypertensive drugs, so the data were not listed in article. Baseline characteristics of the patients among gender, age, and *KCNH2* (1956,C>T) genotypes groups are shown in Table 1.

**Table 1:** Comparison the baseline characteristics of the study population between gender, age, and HERG genotype groups.

Variables	gender		age		Genotype	
	Men(n)	Women(n)	Age≤55 y(n)	Age>55 y(n)	CC(n)	CT+TT(n)
Age, y	57.2±8.9(211)	56.3±7.7(159)	49.3±6.1(156)	62.3±5.0(214)*	56.7±8.7(298)	57.4±7.2(72)
BMI, kg/m^2^	25.4±3.1(211)	24.6±3.9(159)*	25.2±3.1(156)	25.0±3.72(214)	25.2±3.3(298)	24.8±4.0(72)
HR, bpm	75.3±6.8(211)	74.8±8.1(159)	75.3±8.0(156)	74.9±6.9(214)	74.8±7.3(298)	76.3±7.6(72)
SBP, mm Hg	150.0±11.0(211)	149.8±9.5(159)	148.6±10.4(156)	150.8±10.3(214)	149.8±10.3(298)	150.3±10.8(72)
DBP, mm Hg	98.5±4.4(211)	97.5±4.3(159)*	98.6±4.6(156)	97.6±4.2(214)*	98.1±4.5(298)	98.0±3.9(72)
PP, mm Hg	51.4±10.0(211)	52.3±8.7(159)	50.0±9.3(156)	53.1±9.4(214)*	51.7 ±9.3(298)	52.2±10.0(72)
MAP, mm Hg	115.7±5.6(211)	114.9±5.1(159)	115.3±5.6(156)	115.4±5.2(214)	115.3±5.4(298)	115.5±5.2(72)
ALT,umol/L	34.6±25.3(181)	27.5±13.2(136)*	34.3±27.6(143)	29.3±13.7(174)*	32.2±22.7(258)	28.8±12.6(59)
BUN,mmol/L	5.7±5.2(197)	5.2±5.7(142)	4.8±1.4(148)	6.1±7.1(191)*	5.6±6.0(273)	5.3±1.2(66)
UCr, mmol/L	86.9±14.5(208)	69.4±14.8(154)*	76.5±16.1(154)	81.6±17.4(208)*	78.5±17.0(291)	83.0±16.7(71)*
UA, mmol/L	342.7±81.4(129)	273.9±64.7(90)	313.4±85.8(91)	315.2±79.8(128)	312.4±82.6(179)	323.5±80.6(40)
FBG,mmol/L	5.3±1.1(157)	5.2±1.2(118)	5.3±1.2(125)	5.3±1.1(150)	5.3±1.2(225)	5.1±0.7(50)
TG, mmol/	3.9±28.1(208)	1.9±5.4(156)*	2.4±5.6(154)	3.5±27.9(210)	2.0±1.1(293)	7.5±1.3(71)*
CHO,mmol/L	5.2±1.1(208)	5.1±1.2(154)	5.2±1.1(154)	5.2±1.2(208)	5.1±4.2(291)	5.3±48.0(71)
HDL, umol/L	1.3±0.3(197)	1.4±0.3(139)	1.3±0.3(142)	1.4±0.3(194)	1.4±0.3(272)	1.3±0.3(64)
LDL, umol/L	3.3±1.1(83)	3.3±1.2(65)	3.2±1.1(66)	3.4±1.1(82)	3.2±1.2(123)	3.5±0.9(25)

Data expressed as mean±s.d.

* representative P-value (<0.05). BMI indicates body mass index; HR, heart rate; SBP, systolic blood pressure; DBP, diastolic blood pressure; PP, pulse pressure; MAP, mean arterial pressure; ALT, alanine aminotransferase; BUN, blood urea nitrogen, UCr, urine creatinine; UA, uric acid; FBG, fasting blood-glucose; TG, triglyceride; CHO, cholesterol; HDL, high-density lipoprotein; LDL, low density lipoprotein.

### 2. Genotypes and allele frequency

In our study population, the prevalence of *KCNH2* (1956, C>T) polymorphism CC genotype, CT genotype, and TT genotype were 80.5%, 18.6% and 0.9%, respectively. The T allele frequency was 10.1%, and the C allele frequency was 89.9%. This population had no significant deviations in genotype distributions from expected Hardy-Weinberg equilibrium (see table 2).

**Table 2:** Distribution of KCNH2(1956,C>T)genotype and allele frequency in male and female patients.

Genotype	Male(%)	Female(%)	?2	P
CC	170(80.6)	128(80.5)		
CT	39(18.5)	30(18.9)	0.121	0.941
TT	2(0.9)	1(0.6)		
Total	211(57.0)	159(43.0)		

Allele	Male	Female		
C	89.8	89.9	0.396	0.542
T	10.2	10.1		

### 3. Pharmacogenetics study of antihypertensive drugs related to KCNH2 (1956, C>T)

**Figure 1:**
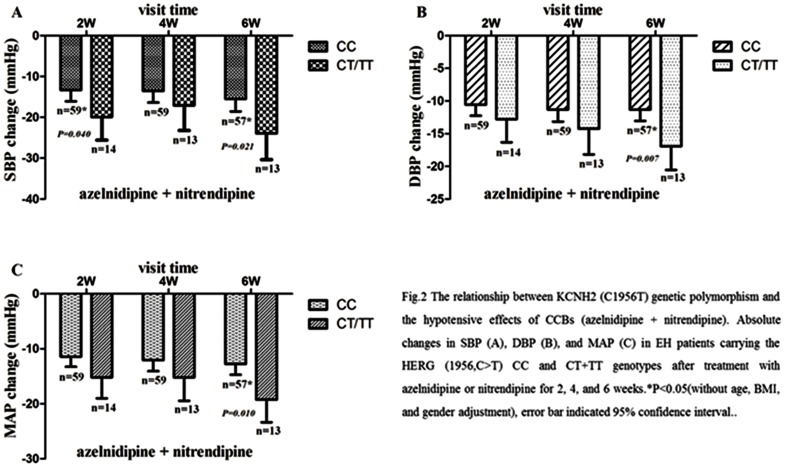
The relationship between *KCNH2* (C1956T) genetic polymorphism and the hypotensive effects of CCBs (azelnidipine & nitrendipine). Absolute changes in SBP (A), DBP (B), and MAP (C) in EH patients carrying the KCNH2 (1956,C>T) CC and CT+TT genotypes after treatment with azelnidipine or nitrendipine for 2, 4, and 6 weeks.*P<0.05(without age, BMI, and gender adjustment), error bar indicated 95% confidence interval.

**3.1 Calcium channel blockers (azelnidipine & Nitrendipine):** The descent of blood pressure after azelnidipine or nitrendipine administration was significantly between KCNH2 (1956,C>T) CC genotype and CT/TT genotype groups (as shown in Figure 1). After adjustment for gender, BMI and age, P-values for DBP change and MAP (mean arterial pressure) change at the end of 6 weeks were 0.010 and 0.014, respectively. While P-values for SBP change at the end of 4 and 6 weeks were 0.193 and 0.059 respectively. Interestingly, our study also found that the relationship between *KCNH2* (1956,C>T) mutation and the effects of CCB is gender-dependent. Significant difference in SBP change followed by age, BMI and Bonferroni correction was observed at the end of 2 weeks (genotype*gender, *P* = 0.028), and a marginally significant difference in MAP change at the end of 6 weeks was also observed (genotype*gender, *P* = 0.060). The results of subgroups stratified by genotype-gender-specific analyses showed that at the end of 2 weeks, significant difference in SBP change between *KCNH2* (1956, C>T) wild type homozygotes and the T allele carriers with Azelnidipine or Nitrendipine therapy was observed only in male patients (Table 3).

**Table 3:** Stratified analyses of the difference hypotensive effects of CCB between KCNH2 (1956,C>T) genotype and gender interaction in EH patients.

variable	men-CC carriers			men-CT/TT carriers			P*(M-CC vs. M-CT/TT)		
	2w	4w	6w	2w	4w	6w	2w	4w	6w
N	28	28	27	7	7	7			
ΔHR(Bpm)	−0.36±9.3	−1.1±8.5	−2.6±8.0	0.3±6.6	−1.1±10.5	−3.7±9.3	1.000	1.000	1.000
ΔSBP(mmHg)	−13.6±9.9	−13.7±8.7	−15.4±10.6	−27.1±13.1	−18.7±16.8	−28.0±16.7	**0.017**	1.000	0.070
ΔDBP(mmHg)	−10.4±5.3	−10.7±6.5	−11.3±7.4	−15.0±7.4	−14.0±9.3	−17..4±6.7	0.515	0.989	0.215
ΔPP(mmHg)	−3.2±7.4	−3.1±7.4	−4.0±9.1	−12.3±8.3	−4.8±10.5	−10.6±11.3	0.095	1.000	0.507
ΔMAP(mmHg)	−11.4±6.2	−11.7±6.4	−12.7±7.5	−19.0±8.8	−15.5±11.3	−21.0±9.7	0.066	1.000	0.067

variable	women-CC carriers			women-CT/TT carriers			P*(W-CC vs. W-CT/TT)		
	2w	4w	6w	2w	4w	6w	2w	4w	6w
N	31	30	30	7	6	6			
ΔHR(Bpm)	−2.6±9.8	−1.8±10.1	−0.9±10.8	0.3±3.3	2.7±5.3	3.5±6.2	1.000	1.000	1.000
ΔSBP(mmHg)	−13.1±10.4	−13.3±12.5	−15.6±11.9	−12.7±7.7	−15.2±7.2	−19.2±5.8	1.000	1.000	1.000
ΔDBP(mmHg)	−10.6±7.5	−11.9±7.9	−11.3±6.1	−10.6±7.1	−14.5±3.0	−16.3±5.1	1.000	1.000	0.442
ΔPP(mmHg)	−2.3±8.1	−1.4±8.6	−4.4±9.0	−2.0±11.0	−0.7±6.1	−2.9±6.0	1.000	1.000	1.000
ΔMAP(mmHg)	−11.4±7.7	−12.4±8.7	−12.8±7.4	−11.3±5.2	−14.8±4.0	−17.2±4.5	1.000	1.000	0.885

Data expressed as mean ± s.d.

* P-value adjustment for BMI and age, multiple comparisons with Bonferroni test; ΔHR = change in heart rate, ΔSBP = change in systolic blood pressure, ΔDBP = change in diastolic blood pressure, ΔPP = change in pulse pressure, ΔMAP = change in mean arterial pressure.

**3.2 α,β-ADR blockers (doxazosin, celiprolol, atenolol & bisoprolol):** As shown in Table 4, patients treated with α,β-ADR blockers(doxazosin, celiprolol, atenolol or bisoprolol monotherapy), a significant *KCNH2* (1956,C>T) genotype specific interaction with age and (or) gender in DBP change and MAP change at the end of 4 weeks and 6 weeks was observed (Table 4). Subgroups stratified by genotype-gender, genotype-age, genotype- gender-age-specific analysis found that the significant differences in DBP change and MAP change in carriers of the *KCNH2* (1956, C>T) CC genotype between male (M-CC) and female (W-CC) patients were observed at the end of 6 weeks. Further study found that the significant differences in DBP and MAP changes between M-CC and W-CC were limited to patients >55 years of age. In addition, when combining doxazosin, atenolol and bisoprolol treatment groups, significant difference in changes in heart rate (HR), DBP and MAP between age≤55y-CC and age>55y-CC group was observed at the end of 4 weeks. Similarly, when combining doxazosin, celiprolol, atenolol and bisoprolol treatment groups, significant difference in changes in HR, DBP and MAP in carriers of the CC genotype between age≤55 y and age>55 y was also observed at the end 4 weeks (Table 5).

**Table 4:** The hypotensive effects of α,β-ADR blockers between KCNH2(C1966T) genotypes(CC v.s CT+TT) and age(≤55 y v.s 55 y), gender(Men v.s Women)interactions in EH patients.

Drugs	Variables(n)	P-value*			Partial Eta Squared			Observed Power		
		gene* gender	gene* age	gene* age* gender	gene* gender	gene* age	gene* age* gender	gene* gender	gene* age	gene* age* gender
doxazosin	ΔDBP6(36)	0.010	0.017	0.027	0.357	0.324	0.416	0.862	0.812	0.821
	ΔMAP6(36)	0.047	0.027	0.036	0.261	0.298	0.401	0.283	0.758	0.791
celiprolol, bisoprolol or atenolol	ΔHR4(91)	<0.001	0.028	0.013	0.267	0.118	0.205	0.997	0.756	0.90
	ΔDBP4(91)	<0.001	0.005	0.002	0.26	0.150	0.254	0.99	0.88	0.971
	ΔMAP4(91)	<0.001	0.030	0.024	0.214	0.116	0.187	0.979	0.749	0.861
doxazosin or celiprolol	ΔDBP6 (89)	0.053	0.054	0.019	0.104	0.104	0.198	0.676	0.675	0.877
	ΔMAP6(89)	0.055	0.165	0.078	0.103	0.074	0.157	0.671	0.492	0.748
doxazosin, bisoprolol or atenolol	ΔHR4(120)	0.023	0.034	0.043	0.095	0.087	0.133	0.778	0.734	0.817
	ΔDBP4(120)	0.002	0.012	0.016	0.141	0.100	0.154	0.94	0.836	0.890
	ΔMAP4(120)	0.007	0.027	0.053	0.115	0.092	0.128	0.870	0.760	0.797
doxazosin, cileprolol, bisoprolol or atenolol	ΔHR4(173)	0.009	0.120	0.093	0.078	0.043	0.079	0.854	0.555	0.739
	ΔDBP4(173	<0.001	0.005	0.007	0.120	0.085	0.124	0.982	0.930	0.892
	ΔMAP4(173)	0.002	0.019	0.035	0.100	0.068	0.096	0.941	0.838	0.793

* P-values were with bonferroni adjust and BMI, gender ,age adjust were appropriately use in the model,ΔHR4 = heart rate change at the end of 4 weeks, ΔDBP4 = diastolic pressure change at the at the end of 4 weeks,ΔMAP4 = mean arterial pressure change at the at the end of 4 weeks,ΔDBP6 = diastolic pressure change at the end of 6weeks, ΔMAP6 = mean arterial pressure change at the end of 6 weeks.

**Table 5:** Stratified analyses of the difference hypotensive effects of α,β-ADR blockers between KCNH2 (1956,C>T) genotypes(CC v.s CT+TT) and age(≤55 y v.s 55 y), gender(Men v.s Women)interactions in EH patients.

Drugs	Genotype, Age and (or) Gender-Specific	variables	mean±S.D	P*-value
doxazosin	M-CC(15) v.s W-CC(13)	ΔDBP6	−13.7±8.3 v.s −0.1±8.6	0.003
		ΔMAP6	−14.7±8.9 v.s −1.9±9.8	0.019
bisoprol or atenolol	CC(34) v.s CT/TT(4)	ΔPP4	−0.8±11.8 v.s −14.0±3.8	0.030
doxazosin or celiprolol	M-CC(36) v.s W-CC(31);	ΔDBP6	−14.1±6.5 v.s −7.5±10.6	0.029
		ΔMAP6;	−15.0±6.9 v.s −8.0±10.4;	0.043 ;
	M-Age>55y-CC (26) v.s W-Age>55y-CC (13)	ΔDBP6	−14.8±4.9 v.s −2.1±11.2	0.004
		ΔMAP6	−15.7±5.3 v.s−3.7±11.2	0.027
doxzosin, bisoprolol or atenolol	Age≤55y-CC (47) v.s Age>55y-CC(47);	ΔHR4	−9.4±14.7 v.s0.7±11.6	0.015
		ΔDBP4	−18.6±10.2 v.s −11.9±8.5	0.008
	Age≤55y-M-CC(25) v.s Age>55y-M-CC(29)	ΔMAP;	−19.6±10.2 v.s −13.2±8.9;	0.029;
		ΔHR4	−9.6±13.6 v.s −2.5±10.9	0.031
cileprolol, bisoprolol or atenolol	Age≤55y-CC v.s Age>55y-CC	ΔDBP4	−20.3±9.3 v.s −13.8±8.2	0.017
		ΔMAP4	−20.7±9.3 v.s −15.0±8.7	0.047
doxazosin, cileprolol, bisoprolol or atenolol	Age≤55y-CC(65) v.s Age>55y-CC(69);	ΔHR4	−6.5±14.0 v.s 0.1±01.2	0.082
		ΔDBP4	−17.2±9.7 v.s−11.8±8.0	0.004
	M-Age≤55y-CC(30) v.s W-Age>55y-CC(24);	ΔMAP4;	−18.1±9.7 v.s−13.2±8.5;	0.023;
		ΔDBP4	18.2±9.7 v.s −10.4±9.0	0.017
	Age≤55y-M-CC(30) v.s Age>55y-M-CC(45)	ΔMAP	−19.4±9.2 v.s −11.9±9.7;	0.048;
		ΔHR4	−8.2±2.4 v.s 0.8±1.4	0.045

* P-values were with bonferroni adjust and BMI, gender ,age adjust were appropriately use in the model,ΔHR4 = heart rate change at the end of 4 weeks, ΔDBP4 = diastolic pressure change at the at the end of 4 weeks,ΔMAP4 = mean arterial pressure change at the at the end of 4 weeks, ΔPP4 = pulse pressure change at the end of 4 weeks, ΔDBP6 = diastolic pressure change at the end of 6weeks, ΔMAP6 = mean arterial pressure change at the end of 6 weeks.

## Discussion

Our study indicates that the hypotensive effects of azelnidipine and nitrendipine are more sensitive in T allele carriers than wild-type carriers of *KCNH2* (1956, C>T) in EH patients. We also observed that the association of which the hypotensive effect of azelnidipine or nitrendipine is genotype-gender dependent. Literature reported that the sensitivity of doxazosin [19] and W-7 (an inhibition of calmodulin) [24] to *KCNH2* potassium channels are decreased due to the mutations like Y652A, F656A in the *KCNH2* pore-S6 region. Zhang et al. [25] reported for verapamil caused high-affinity block of KCNH2 channel current is close to its block of L-type Ca2+ channels', whereas diltiazem only weakly suppresses KCNH2 current, and nifedipine has no effect. These studies suggest that the direct actions of dihydropyridine CCBs on *KCNH2* potassium channels are less likely. Meanwhile, antioxidant stress approach [26] of CCB may play a pivotal role in increasing the activity of *KCNH2* potassium channels [5]. So, according to our results, we can assume that the ability of azelnidipine/nitrendipine and its downstream semiochemical may interact with variant *KCNH2* channels more weakly than the wild-type channels. Then the excitability and contractibility inhibited by CCB on variant allele carriers of *KCNH2* potassium channels are more sensitive in smooth muscles [2], and eventually, the hypotensive effects of azelnidipine or nitrendipine on EH patient's therapy is more apparent in carriers of the *KCNH2(*1956,C>T) T allele.

**Figure 2:**
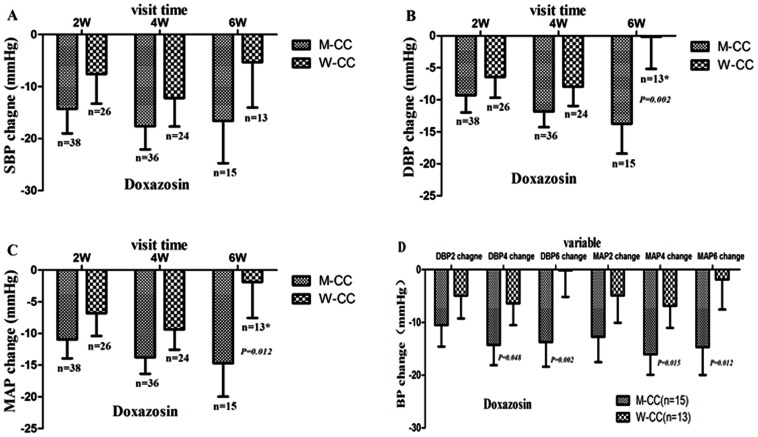
*KCNH2* (1956,C>T) genetic polymorphism in the response to doxazosin is gender-specific. A,B,C depicts that the changes in systolic blood pressure, diastolic blood pressure and mean arterial pressure in men-CC genotype carriers v.s women-CC genotype carriers with essential hypertension after 2 weekend,4 weekend and 6 weekend follow-up to those who were medications with doxazosin. D showed that all follow-up records have no missing at the end of 2,4 and 6 weeks and the polymorphism of KCNH2(C195T) related to the effects of doxazosin is gender-specific,*P<0.05 as compared with corresponding men-CC.

Unexpectedly, our study shows that the SBP changes between *KCNH2* (1956,C>T) CC and CT+TT genotypes are significantly different at the end of 2 and 6 weeks but not at the end of 4 weeks after azelnidipine or nitrendipine therapy (see Fig 2 and table 2). Studies have shown that the modest fluctuations of Ca^2+^ concentration may lead to marked changes on *KCNH2* K^+^ current: a reduction of *I_kr_* in result of external Ca^2+^ elevation was not due to Ca^2+^ acting as a direct blocker of the open pore, but as an allosteric modulator of the *KCNH2* potassium channels [27], [28]. However, Koyama et al. [29] found that CCBs have an excellent antioxidant stress effect, which subsequently increase the activity of *KCNH2* potassium channels [5]. From the studies above, the phenomenon may be intrigued by the dynamic regulation of calcium channels blocking effect and the antioxidant stress effect of azelnidipine or nitrendipine on *KCNH2* potassium channels: the effects of azelnidipine or nitrendipine antioxidant stress pathway can account for T allele carriers, while the calcium channels blocking effect may be a better explanation for CC genotype carriers, both of which should be studied intensively.

As summarized in table 4 and Table 5, the *KCNH2* (1956,C>T) polymorphism is associated with the effects of α,β-ADR blockers in a genotype-age-gender dependent manner. Interestingly, stratified analyses according to the interactions between genotype and age, gender were observed, the significantly different effects of α,β-ADR blockers on EH patients only exist in *KCNH2* (1956,C>T) CC*gender-, CC*age- or CC*gender*age-specific groups. The hypotensive effects of β-ADR blockers are more obvious in age≤55y-CC than age>55-CC genotype carriers, and a significant difference in PP change between CC and CT+TT genotypes at the end of 4 weeks was also observed. However, α-ADR blocker in the men CC genotype carriers group is more sensitive than women group (see Figure 2). When analyzing combined α,β-ADR blockers, we found that the effects in men CC genotype carriers group is better than women, so does it between age≤55y-CC and age>55y-CC genotype carriers, but there is no significant difference in T allele carriers between the group.

Studies have shown that activating PKA and cAMP in endoplasmic reticulum surface can up-regulate the expression of *KCNH2* [30], [31]. Progesterone and β-estradiol which can induce the trafficking defect of KCNH2 [32], [33], [34]. While, dihydrotestosterone can repair damaged *KCNH2* and improve its expression [35]. Moreover, the expression of *KCNH2* mRNA in females is lower than that in males, and significant difference in *KCNH2* expression between age<55 y and age>55 y group in LQTS patients is also reported [36]. Additionally, Thomas et al. [19] reported that doxazosin can directly block *KCNH2*, and the sensitivity is influenced by the mutation of *KCNH2* (1956,C>T), while other research found that celiprolol involved in modulating the function of endothelial cells and had certain antioxidant stress effects [37], [38]. Furthermore, β-ADR blockers possess weak affinity with *KCNH2* potassium channels, while atenolol did not block *I_kr_* current [30], [31], and β1-ADR blockers (α1-ADR blockers) can be involved in regulating the expression of α1-ADR (β1-ADR) directly or indirectly [39]. Together, the aforementioned studies may provide us some clues to the reason why the *KCNH2* (1956,C>T) polymorphism is related to the genotype-age-gender dependent effects of α,β-ADR blockers, and the high sensitivity of α,β-ADR blockers in men or age<55 y group.

We also found that the *KCNH2* (1956,C>T) was related to HR change (P = 0.035) rather than BP change at the end of 6 weeks between age≤55 yCC(n = 36) and age>55yCC(n = 62) medicated with imidapril, candesartan or irbesartan. Irbesartan and candesartan could decrease the QT interval in hypertension patients due to the increased *I_kr_* currents [40], [41]. The inhibition of *I_kr_* was realized via its phosphorylation by ANGII–AT1R–PKC pathway or Ang II directly interact with *KCNH2*, and that was not influenced by the change of Ca^2+^ concentration or PKA pathway. This indicates that it may activate a special subtype of PKC [42], which may be a leading cause to interpret why the *KCNH2*(1956,C>T) polymorphism is not associated with the response to imidapril, candesartan or irbesartan in EH patents.

At present, only a few literatures have reported the mechanisms of these pathways, but its exact mechanism is still unclear. Our study indicated that the function of KCNH2 potassium channels,which regulated by α,β-ADR, oxidative stress, regulator protein (Caveolin-1), effectors (PIP2,PKA/PKC) mediated pathways may through interacting with the pore-S6 region. Furthermore, the KCNH2 (1956C>T) polymorphism dependent on age, as well as gender difference is important to determinate the sensibility of drugs, which are involved in modulating the expression or the properties of KCNH2 potassium channels. Interestingly, our results are in accordance with the *in vitro* functional studies for KCNH2 (1956C>T) mutation. Hence, we conclude that the KCNH2 (1956,C>T) polymorphism may serve as a novel biomarker for prediction of the response to CCBs and α,β-ADR blockers in EH patients.

## Materials and Methods

### Patients and study design

The study protocol was approved by the Ethical Committee of the Institute of Clinical Pharmacology, Central South University, China. The registration number (ChiCTR-RO-12002612) was validated by the Chinese Clinical Trial Registry, and written informed consent was obtained from all patients prior to study entry. The clinical data and DNA samples were graciously provided by Shanghai Institute of Hypertension, Ruijin Hospital affiliated with Shanghai Jiaotong University. This study was an open label clinical trial, in which a total of 453 eligible patients was enrolled after a run-in period of 2 weeks and assigned to receive the drugs for 4 weeks or more, and details of the protocol were shown in Table 6. Blood pressure determinations were performed in the morning after a light breakfast with subjects in the seated position, and following a 30 min quiet resting period. Blood pressure and heart rate were measured by trained nurses, with an automatic blood pressure monitor with intellisense, which allows the detection of alteration of the heart rate by greater than or equal to beat/min and of the blood pressure by greater than or equal to 1 mmHg. Monitors were validated against a mercury sphygmomanometer. The blood pressure values were determined as the average of three measurements taken 10 min apart. Values for SBP and DBP were defined by Korotkoff phase I and V, respectively. Pulse pressure was calculated as PP = (SBP- DBP); mean arterial pressure(MAP) was calculated as MBP = DBP+(PP/3).

**Table 6:** The protocols of the related drugs to a total of 453 eligible patients in the study.

Drugs & Dose	simple size(N)	treatment course(W)	case report
celiprolol (200 mg/d)	55	6	ETW
atenolol(25 mmg/d)	21	4	EQW
bisoprolol(5 mmg/d)	31	4	EQW
doxazsin(2 mmg/d)	91	6	ETW
azelnidipine(2 mmg/d)	67	6	ETW
nitrendipine+atenolol(5+10 mmg/d	36	6	ETW
irbesartan(150 mmg/d)	14	6	ETW
candesartan(8 mmg/d)	39	6	ETW
imidapril(5 mmg/d)	88	6 or 8	ETW or EQW
olmesartan+amlodipine(20+5 mmg/d)	3	8	EQW
benazepril+hydrochlorothiazide (5+6.25 mmg/d)	8	8	EQW

N = number; W = week; ETW = every two weeks; EQW = every four weeks.

**Figure 3:**
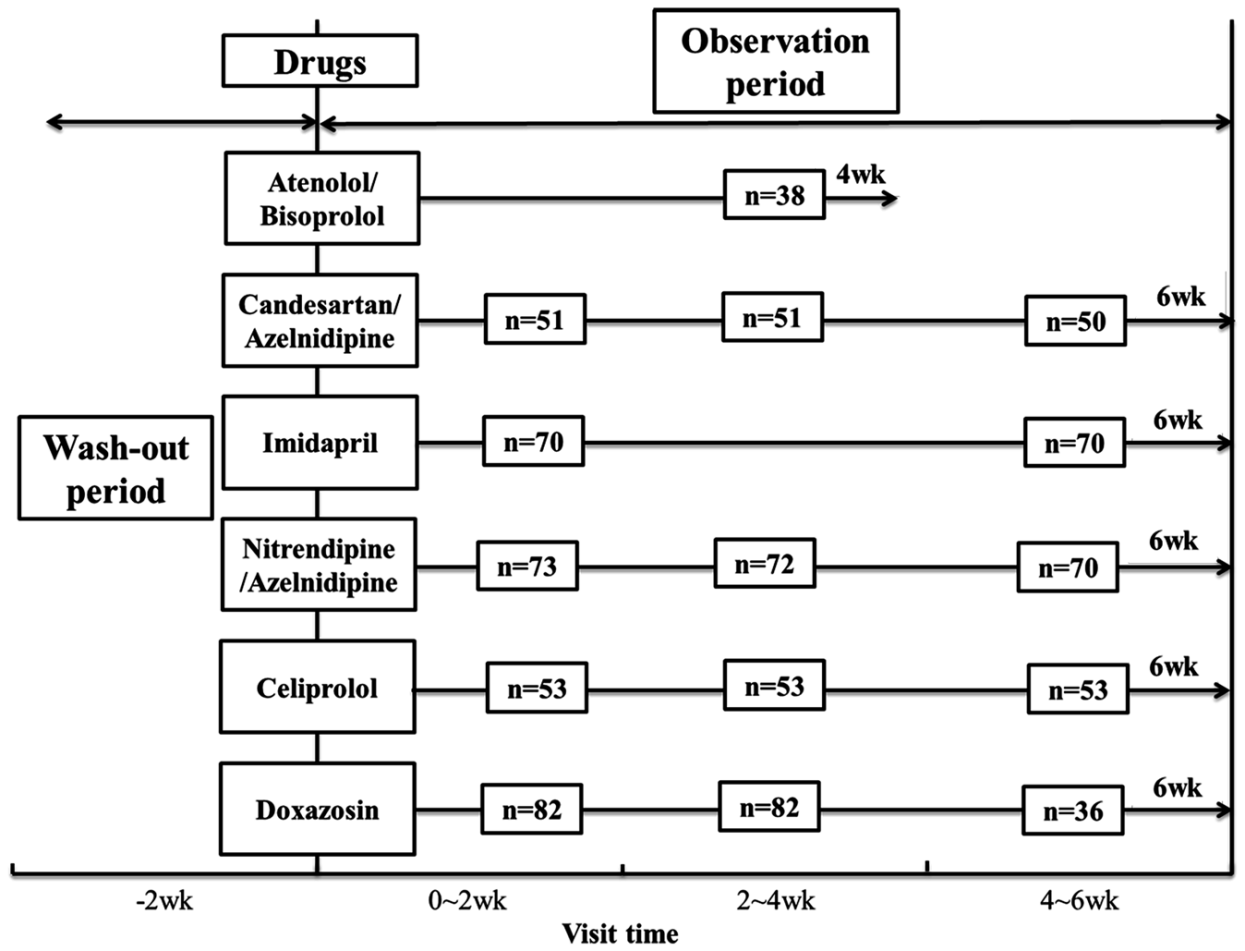
Study protocol and visit time for the 370 patients treated with corresponding drugs in the study; wk: week.

According to our purpose, all the patients fulfilled the following inclusion and exclusion criteria: male or female patients, heart rates within 55∼90 beats/min, SBP≥140 mmHg and/or DBP≥90 mmHg were included. Patients with secondary hypertension, coronary heart disease, diabetes, obesity (BMI>30 kg/m^2^), stroke, renal or liver dysfunction, malignant tumor or pregnancy women and those whose blood pressure measurements above 180/110 mmHg or remaining lower than 140/90 mmHg during the wash-out period, were withdrawn from the study. Finally, 83 samples were excluded from 453 eligible patients due to the small sample size, loss to follow-up and unqualified g-DNA. So, 370 patients were included in our study. The medications include β-ADR blockers (atenolol, bisoprolol and celiprolol), α-ADR block (doxazosin), CCBs (azelnidipine and nitrendipine), ACEI (Imidapril) and AT1R blockers (Candesartan and irbesartan). Details were shown in Figure 3.

### Genotyping

Sequencing in 85 samples in the frequency of *KCNH2* rs8179011 (1966–1967insA/T) was not reported in Chinese population. Genotyping of *KCNH2* rs1137617 (1956 C>T) polymorphisms were verified by sanger sequencing and polymerase chain reaction and restriction fragment length polymorphism assays (PCR-RFLP).The PCR primers used in the amplification were: 5′TACAAGGG CTCTGTGG3′ (forward) and 5′TGGTGGAAGC GGATGAAC3′ (reverse). The oligonucleotide primers were synthesized by Invitrogen Trading (shanghai) Co., Ltd. The optimized PCR system were carried out with a total volume of 25 ul composed of 10x PCR buffer (2.5 ul), 10x dNTP (2.5 ul), 10 uM for each of the forward and reverse primers (0.5 ul), H_2_O (16.8 ul), g - DNA (2 ul), Taq-ase (0.2 ul), and it was performed for thirty-six cycle of amplification: denaturation at 94°C for 30 s, annealing at 52.8°C for 30 s, and elongation at 72°C for 30 s. While an initial denaturation step was implemented at 94°C for 5 min, a final elongation at 72°C for 5 min. All the processes forenamed were carried out with silver tank PCR instrument (eppendorf AG, Germany). Digestion of the PCR products with *Csp6* I (Fermentas) was carried out according to the criteria. The digestion products were analyzed by gel electrophoresis using 2.5% agarose (gene tech. company, Shanghai, China), sequencing were assisted by Shanghai Majorbio Bio-pharm Technology Company. KCNH2 rs1805123(2690,A>C) genotyping was carried out as described by Bezzina et al.[21]. A modified amplification system composed of 2xGC buffer(12.5 ul),10x dNTP (2.5 ul),10 uM sense and anti-sense primer (1 ul), H_2_O (5.8 ul), g-DNA (2 ul), Taq-ase (0.2 ul) was used. The amplification conditions were as follows: initial denaturation at 94°C for 5 min, followed by 36 cycles of 94°C for 30 s, 61.8°C for 30 s, 72°C for 30 s, and then a final elongation at 72°C for 5 min.

### Statistical Analysis

All data were presented as mean values ±S.D unless otherwise specified. Difference in the baseline characteristics between genders (men v.s women), KCNH2(1956,C>T) genotypes(CC v.s CT+TT), and ages(age≤55 y v.s age>55) were compared by independent-samples T-test or Wilcoxon rank sum test, appropriately. Hardy-Weinberg equilibrium for genotypic distribution of KCNH2 (1956, C>T) polymorphism was analyzed by using χ2 test. Allele frequencies were determined by direct gene counting. The differences between groups of the changes (after treatment-before treatment) in HR, SBP DBP, MAP, and PP were calculated using multivariable ANOVA analysis followed by Bonferroni correction for multiple comparisons. P- values were adjusted by BMI, gender and age when needed. To address whether significant interaction between age-genotype, gender-genotype, age-gender-genotype specific and the response on antihypertensive therapy, multivariate ANOVA and Stratified Analysis were used. A two-tailed P-value<0.05 was considered significant. Statistical analyses were performed using SPSS 19.0 for Windows software (SPSS, Chicago, IL).

## References

[1] Chobanian AV, Bakris GL, Black HR, Cushman WC, Green LA, et al (2003) The Seventh Report of the Joint National Committee on Prevention, Detection, Evaluation, and Treatment of High Blood Pressure: the JNC 7 report. JAMA 289: 2560–2572. 12748199 10.1001/jama.289.19.2560

[2] Akbarali HI, Thatte H, He XD, Giles WR, Goyal RK (1999) Role of HERG-like K(+) currents in opossum esophageal circular smooth muscle. Am J Physiol 277: C1284–C1290. 10600781 10.1152/ajpcell.1999.277.6.C1284

[3] Arcangeli A, Bianchi L, Becchetti A, Faravelli L, Coronnello M, et al (1995) A novel inward-rectifying K+ current with a cell-cycle dependence governs the resting potential of mammalian neuroblastoma cells. J Physiol 489 (Pt 2) 455–471. 8847640 10.1113/jphysiol.1995.sp021065 PMC1156772

[4] Taglialatela M, Castaldo P, Iossa S, Pannaccione A, Fresi A, et al (1997) Regulation of the human ether-a-gogo related gene (HERG) K+ channels by reactive oxygen species. Proc Natl Acad Sci U S A 94: 11698–11703. 9326673 10.1073/pnas.94.21.11698 PMC23597

[5] Jury J, Boev KR, Daniel EE (1996) Nitric oxide mediates outward potassium currents in opossum esophageal circular smooth muscle. Am J Physiol 270: G932–G938. 8764199 10.1152/ajpgi.1996.270.6.G932

[6] Wang S, Xu DJ, Cai JB, Huang YZ, Zou JG, et al (2009) Rapid component I(Kr) of cardiac delayed rectifier potassium currents in guinea-pig is inhibited by alpha(1)-adrenoreceptor activation via protein kinase A and protein kinase C-dependent pathways. Eur J Pharmacol 608: 1–6. 19233158 10.1016/j.ejphar.2009.02.017

[7] Tutor AS, Delpon E, Caballero R, Gomez R, Nunez L, et al (2006) Association of 14-3-3 proteins to beta1-adrenergic receptors modulates Kv11.1 K+ channel activity in recombinant systems. Mol Biol Cell 17: 4666–4674. 16914520 10.1091/mbc.E06-05-0422 PMC1635398

[8] Krishnan Y, Li Y, Zheng R, Kanda V, McDonald TV (2012) Mechanisms underlying the protein-kinase mediated regulation of the HERG potassium channel synthesis. Biochim Biophys Acta 1823: 1273–1284. 22613764 10.1016/j.bbamcr.2012.05.012 PMC3398701

[9] Acosta E, Mendoza V, Castro E, Cruzblanca H (2007) Modulation of a delayed-rectifier K+ current by angiotensin II in rat sympathetic neurons. J Neurophysiol 98: 79–85. 17493917 10.1152/jn.01103.2006

[10] Wang YH, Shi CX, Dong F, Sheng JW, Xu YF (2008) Inhibition of the rapid component of the delayed rectifier potassium current in ventricular myocytes by angiotensin II via the AT1 receptor. Br J Pharmacol 154: 429–439. 18414380 10.1038/bjp.2008.95 PMC2442448

[11] Chun YS, Shin S, Kim Y, Cho H, Park MK, et al (2010) Cholesterol modulates ion channels via down-regulation of phosphatidylinositol 4,5-bisphosphate. J Neurochem 112: 1286–1294. 20015154 10.1111/j.1471-4159.2009.06545.x PMC2891522

[12] Rebsamen MC, Church DJ, Morabito D, Vallotton MB, Lang U (1997) Role of cAMP and calcium influx in endothelin-1-induced ANP release in rat cardiomyocytes. Am J Physiol 273: E922–E931. 9374678 10.1152/ajpendo.1997.273.5.E922

[13] Best JM, Foell JD, Buss CR, Delisle BP, Balijepalli RC, et al (2011) Small GTPase Rab11b regulates degradation of surface membrane L-type Cav1.2 channels. Am J Physiol Cell Physiol 300: C1023–C1033. 21248079 10.1152/ajpcell.00288.2010 PMC3093944

[14] Zhou Q, Bett GC (2010) Regulation of the voltage-insensitive step of HERG activation by extracellular pH. Am J Physiol Heart Circ Physiol 298: H1710–H1718. 20363888 10.1152/ajpheart.01246.2009

[15] Lin J, Lin S, Choy PC, Shen X, Deng C, et al (2008) The regulation of the cardiac potassium channel (HERG) by caveolin-1. Biochem Cell Biol 86: 405–415. 18923542 10.1139/o08-118

[16] Sun T, Guo J, Shallow H, Yang T, Xu J, et al (2011) The role of monoubiquitination in endocytic degradation of human ether-a-go-go-related gene (hERG) channels under low K+ conditions. J Biol Chem 286: 6751–6759. 21177251 10.1074/jbc.M110.198408 PMC3057800

[17] Massaeli H, Sun T, Li X, Shallow H, Wu J, et al (2010) Involvement of caveolin in low K+-induced endocytic degradation of cell-surface human ether-a-go-go-related gene (hERG) channels. J Biol Chem 285: 27259–27264. 20605793 10.1074/jbc.M110.124909 PMC2930725

[18] Sanguinetti MC, Tristani-Firouzi M (2006) hERG potassium channels and cardiac arrhythmia. Nature 440: 463–469. 16554806 10.1038/nature04710

[19] Thomas D, Wimmer AB, Wu K, Hammerling BC, Ficker EK, et al (2004) Inhibition of human ether-a-go-go-related gene potassium channels by alpha 1-adrenoceptor antagonists prazosin, doxazosin, and terazosin. Naunyn Schmiedebergs Arch Pharmacol 369: 462–472. 15098086 10.1007/s00210-004-0931-8

[20] Aiba T, Hesketh GG, Liu T, Carlisle R, Villa-Abrille MC, et al (2010) Na+ channel regulation by Ca2+/calmodulin and Ca2+/calmodulin-dependent protein kinase II in guinea-pig ventricular myocytes. Cardiovasc Res 85: 454–463. 19797425 10.1093/cvr/cvp324 PMC2802203

[21] Guo J, Li X, Shallow H, Xu J, Yang T, et al (2011) Involvement of caveolin in probucol-induced reduction in hERG plasma-membrane expression. Mol Pharmacol 79: 806–813. 21278233 10.1124/mol.110.069419

[22] Sarzani R, Pietrucci F, Corinaldesi C, Francioni M, Letizia C, et al (2006) The functional HERG variant 897T is associated with Conn's adenoma. J Hypertens 24: 479–487. 16467651 10.1097/01.hjh.0000209984.28735.fd

[23] Oshiro C, Thorn CF, Roden DM, Klein TE, Altman RB (2010) KCNH2 pharmacogenomics summary. Pharmacogenet Genomics 20: 775–777. 20150828 10.1097/FPC.0b013e3283349e9c PMC3086352

[24] Zhang XH, Jin MW, Sun HY, Zhang S, Li GR (2010) The calmodulin inhibitor N-(6-aminohexyl)-5-chloro-1-naphthalene sulphonamide directly blocks human ether a-go-go-related gene potassium channels stably expressed in human embryonic kidney 293 cells. Br J Pharmacol 161: 872–884. 20860665 10.1111/j.1476-5381.2010.00916.x PMC2992901

[25] Zhang S, Zhou Z, Gong Q, Makielski JC, January CT (1999) Mechanism of block and identification of the verapamil binding domain to HERG potassium channels. Circ Res 84: 989–998. 10325236 10.1161/01.res.84.9.989

[26] Koyama Y, Takeishi Y, Takahashi H, Shishido T, Arimoto T, et al (2007) Azelnidipine inhibits H2O2-induced cell death in neonatal rat cardiomyocytes. Cardiovasc Drugs Ther 21: 69–72. 17318380 10.1007/s10557-007-6008-4

[27] Johnson JJ, Balser JR, Bennett PB (2001) A novel extracellular calcium sensing mechanism in voltage-gated potassium ion channels. J Neurosci 21: 4143–4153. 11404399 10.1523/JNEUROSCI.21-12-04143.2001 PMC6762739

[28] Johnson JJ, Mullins FM, Bennett PB (1999) Human ether-a-go-go-related gene K+ channel gating probed with extracellular ca2+. Evidence for two distinct voltage sensors. J Gen Physiol 113: 565–580. 10102937 10.1085/jgp.113.4.565 PMC2217168

[29] Koyama Y, Takeishi Y, Takahashi H, Shishido T, Arimoto T, et al (2007) Azelnidipine inhibits H2O2-induced cell death in neonatal rat cardiomyocytes. Cardiovasc Drugs Ther 21: 69–72. 17318380 10.1007/s10557-007-6008-4

[30] Dupuis DS, Klaerke DA, Olesen SP (2005) Effect of beta-adrenoceptor blockers on human ether-a-go-go-related gene (HERG) potassium channels. Basic Clin Pharmacol Toxicol 96: 123–130. 15679475 10.1111/j.1742-7843.2005.pto960206.x

[31] Sroubek J, McDonald TV (2011) Protein kinase A activity at the endoplasmic reticulum surface is responsible for augmentation of human ether-a-go-go-related gene product (HERG). J Biol Chem 286: 21927–21936. 21536683 10.1074/jbc.M110.201699 PMC3122247

[32] Ueno K, Sato H (2012) Gender-related differences in pharmacokinetics and pharmacodynamics of anti-hypertensive drugs. Hypertens Res 35: 245–250. 22089536 10.1038/hr.2011.189

[33] Wu ZY, Yu DJ, Soong TW, Dawe GS, Bian JS (2011) Progesterone impairs human ether-a-go-go-related gene (HERG) trafficking by disruption of intracellular cholesterol homeostasis. J Biol Chem 286: 22186–22194. 21525004 10.1074/jbc.M110.198853 PMC3121363

[34] Ando F, Kuruma A, Kawano S (2011) Synergic effects of beta-estradiol and erythromycin on hERG currents. J Membr Biol 241: 31–38. 21484263 10.1007/s00232-011-9360-z

[35] Ridley JM, Shuba YM, James AF, Hancox JC (2008) Modulation by testosterone of an endogenous hERG potassium channel current. J Physiol Pharmacol 59: 395–407. 18953086

[36] Moric-Janiszewska E, Glogowska-Ligus J, Paul-Samojedny M, Weglarz L, Markiewicz-Loskot G, et al (2011) Age-and gender-dependent mRNA expression of KCNQ1 and HERG in patients with long QT syndrome type 1 and 2. Arch Med Sci 7: 941–947. 22328875 10.5114/aoms.2011.26604 PMC3264984

[37] Hattori K, Yamanouchi D, Banno H, Kobayashi M, Yamamoto K, et al (2007) Celiprolol reduces the intimal thickening of autogenous vein grafts via an enhancement of nitric oxide function through an inhibition of superoxide production. J Vasc Surg 46: 116–123. 17606127 10.1016/j.jvs.2007.03.044

[38] Yao EH, Fukuda N, Matsumoto T, Katakawa M, Yamamoto C, et al (2008) Effects of the antioxidative beta-blocker celiprolol on endothelial progenitor cells in hypertensive rats. Am J Hypertens 21: 1062–1068. 18636069 10.1038/ajh.2008.233

[39] Mizuno K, Kurokawa K, Shibasaki M, Ohkuma S (2011) beta(1)-adrenergic receptor up-regulation induced by nadolol is mediated via signal transduction pathway coupled to alpha(1)-adrenergic receptors. Brain Res 1414: 10–21. 21871614 10.1016/j.brainres.2011.07.057

[40] Moreno I, Caballero R, Gonzalez T, Arias C, Valenzuela C, et al (2003) Effects of irbesartan on cloned potassium channels involved in human cardiac repolarization. J Pharmacol Exp Ther 304: 862–873. 12538844 10.1124/jpet.102.042325

[41] Caballero R, Delpon E, Valenzuela C, Longobardo M, Gonzalez T, et al (2001) Direct effects of candesartan and eprosartan on human cloned potassium channels involved in cardiac repolarization. Mol Pharmacol 59: 825–836. 11259627 10.1124/mol.59.4.825

[42] Wang YH, Shi CX, Dong F, Sheng JW, Xu YF (2008) Inhibition of the rapid component of the delayed rectifier potassium current in ventricular myocytes by angiotensin II via the AT1 receptor. Br J Pharmacol 154: 429–439. 18414380 10.1038/bjp.2008.95 PMC2442448

